# Reporting renal biopsies from Cyprus: a systematic approach

**DOI:** 10.15171/jnp.2017.38

**Published:** 2017-04-13

**Authors:** Düriye Deren Oygar, Guy H. Neild

**Affiliations:** ^1^Department of Nephrology, Nicosia State Hospital, Burhan Nalbantoglu General Hospital, North Cyprus; ^2^UCL Centre for Nephrology, University College London, London NW3 2QG, UK

**Keywords:** Primary renal disease, Renal biopsy, Renal failure, Middle East, Chronic kidney disease

## Abstract

**Background:**

The etiology of renal disease varies in different parts of the world. In the Middle East, half of all patients reaching end-stage are categorised as either unknown etiology or hypertension-related nephropathy.

**Objectives:**

To report a renal biopsy series, in a reproducible format and manner, so that data can be compared directly among other series.

**Patients and Methods:**

Biopsies of native kidneys were performed in a 10-year period, at a tertiary referral hospital that provides the entire nephrology service for north Cyprus. Data are reported from 153 patients older than 17 years, who were either Turkish-Cypriot or from the Turkish mainland.

**Results:**

Mean biopsy rate was 48 per million population (pmp) per year. Mean age was 45.7 years (range 18-78). Overall, the sex distribution was similar (male 51%). The most common histopathological categories were primary glomerulonephritis (GN) (56%), secondary GN (27%), and tubulo-interstitial disease (14%). Of those with primary GN, 29% had secondary (2o) focal and segmental glomerulosclerosis (FSGS) (29%), followed by IgA nephropathy (24 %), membranous 18% and a further 11 patients with 1o FSGS (12%). The incidence of IgA nephropathy was 6.3 per pmp/year. When expressed as a percentage of the annual biopsy rate, 14% of all biopsies showed IgA nephropathy.

**Conclusions:**

To compare data among centres, they must be expressed in terms of the population (incidence pmp/year) and the biopsy rate. In our population, secondary FSGS is common and uncharacterised and we believe many will be caused by monogenic disease.

**Implication for health policy/practice/research/medical education:**

Evidence from the island of Cyprus suggests that renal failure is much more common than in western Europe. This is partly owing to the high prevalence of familial renal disease and partly the burden of diabetes. The epidemiology of renal disease throughout the Middle East is likely to be similar to Cyprus. To make progress in understanding renal disease, biopsy data must be reported in a reproducible and systematic manner so that meaningful comparisons can be made amongst different centres. Diabetic patients should be biopsied when the clinical renal findings are not consistent with diabetic nephropathy. It is recommended that processing and reporting of renal biopsies is concentrated in centres able to perform immunoperoxidase immunostaining on formalin-fixed tissue.

## 1. Background

Renal biopsies are important for making a diagnosis but also for guiding treatment and prognosis. The current classification of glomerular and tubular disease was established by a committee under the auspices of the World Health Organization (WHO) in 1982. Diagnostic groups were defined, based on biopsy material principally from West Europe, USA and Japan which had been examined by light microscopy, immunofluorescence (IF), and electron microscopy (EM) (1). Since then it is generally assumed that there is little more to know but is this true? Can we still learn more about diagnosis from biopsies?

It is common for renal centres to compare their biopsy series with others and speculate on regional, geographical and ethnic differences but, because there is no standardisation of presentation, like is often not compared with like. For example, some series include children, some transplant biopsies, some just glomerular disease. Criteria for biopsy vary, some centres do not biopsy diabetics, some do not biopsy those without proteinuria, some biopsy everyone with persistent microscopic haematuria. Furthermore, without definition the following commonly used terms are either meaningless or at best ambiguous; diabetic kidney disease, focal and segmental glomerulosclerosis (FSGS), nephrotic syndrome (NS), hypertension/hypertensive nephropathy.

In the Middle East there is still much to learn; at least 50% of patients with end-stage kidney disease have no clear diagnosis (2), and in the past decade several new diseases have been described (3-6).

## 2. Objectives

To report a renal biopsy series, in a reproducible format and manner, so that data can be compared directly among other series. By reviewing our own small series, we will highlight the issues that must be addressed and areas for further research.

## 3. Patients and Methods

### 3.1. Study population

This is a retrospective study of renal biopsies performed, in the period January 2006 through 2015, at a tertiary referral hospital that provides the nephrology service for the entire population of north Cyprus in a single centre. All research involving human participants was performed with written informed consent and was approved by the ethics committee of Lefkosa Burhan Nalbantoğlu State Hospital. Biopsies of native kidneys were performed on 153 adult patients older than 17 years, who were either Turkish-Cypriot or from the Turkish mainland. The community, over this period, represents a population of approximately 320 000 people aged 17 years or older (7). Biopsies from other patients, and all transplant biopsies, were excluded. Demographic characteristics, clinical presentation, indications for renal biopsy, pathological diagnosis of all the patients biopsied were recorded, together with presence of hypertension and/or antihypertensive treatment, serum creatinine and albumin, proteinuria (g/d), and urinary sediment. We also noted the main renal syndrome.

Renal biopsy specimens were evaluated by light microscopy, and direct immuno-fluorescence (IgG, IgA, IgM, C3, C1q, fibrinogen, and light-chain antibodies) in 91% of cases. Electron microscopic evaluation was done in 28 biopsies (18%).

Clinical and histopathological classifications are summarised from similar studies (see Tables 1-2) (8-12).

### 3.2. Definition of clinical terms

Nephrotic syndrome (NS) describes patients with heavy proteinuria, greater than 3 g/d (300 mg/mmol creatinine or 3000 mg/g), and with hypoproteinaemia defined as a serum albumin below the normal range. The majority will have dependent oedema.

Asymptomatic urinary abnormalities (AUA) includes patients with proteinuria and/or persistent microscopic hematuria without symptoms; with or without chronic renal failure.

Patients with proteinuria >3 g/d and normal serum albumin, are categorised in the group of AUAs.

Chronic tubular kidney disease refers to those with chronic kidney disease (CKD) (estimated glomerular filtration rate [eGFR] <60 mL/min) but little or no proteinuria (<1 g/d). Thus, this represents a group with a clinical phenotype of tubular disease. Those with CKD and urinary abnormalities are reported in the group AUA.

Primary FSGS (1^o^ FSGS) describes patients with NS, which may or may not be steroid-sensitive, and whose biopsy shows features of FSGS.

Secondary FSGS (2^o^ FSGS) is used to refer to a miscellaneous group of patients whose cause of renal failure is rarely known and who have a normal serum albumin. For example, patients with renal dysplasia/reflux nephropathy and evidence of scarring by DMSA scan may develop 2^o^ FSGS. To the best of our knowledge, none of those biopsied had evidence of asymmetric kidneys (by renal ultrasound or DMSA scan) that was consistent with a diagnosis of either reflux nephropathy or reno-vascular disease.

Diabetic nephropathy: renal biopsy shows principally the typical features of diabetic glomerular pathology.

### 3.3. Definition of renal phenotypes

Clinical indications for renal biopsies were classified as:
• Glomerular phenotype
1) NS: proteinuria ≥3.0 g/d (>300 mg/mmol creatinine) with hypoalbuminaemia (albumin below normal).
2) AUA: proteinuria and/or persistent microscopic hematuria without symptoms; with or without CRF.
3) Acute nephritic syndrome: includes acute fall in GFR, edema, hematuria, proteinuria and hypertension.
• Tubular phenotype
4) Chronic tubular kidney disease: persistent eGFR <60 mL/min with minimal or no proteinuria (<1 g/d).
5) Acute kidney injury (AKI): rapid deterioration of GFR, including worsening of CKD; with bland urinary sediment.

### 3.4. Histopathological classification

According to biopsy results, renal diseases were categorised pathologically into five broad groups as:

A) Primary glomerulonephritides (GN),
B) Secondary GN,
C) Tubulointerstitial disease (TID),
D) Vascular nephropathies (VN) and
E) Others (end-stage kidney disease, inadequate size).

The subdivisions of these groups are shown in Table 1.

**Table 1:** Histopathological classification

A. Primary glomerulonephritis (GN):
1. Minimal change disease (MCD)
2. Primary Focal and segmental glomerulosclerosis (1^o^FSGS)
3. Secondary Focal and segmental glomerulosclerosis (2^o^FSGS)
4. Diffuse endocapillary GN (with immunological deposits in biopsy)
5. Crescentic/necrotising GN in the absence of systemic disease:
I. type 1 (accompanied by anti-glomerular basal membrane antibodies),
II. type 2 (presence of immune complexes), and
III.type 3 [necrotizing GN with or without anti-neutrophil cytoplasmic antibodies (ANCAs)
6. Membranoproliferative GN type 1, and 2 (dense deposit disease) (MPGN)
7. Membranous nephropathy (MN)
8. IgA nephropathy (IgAN)
9. Non-IgA mesangioproliferative GN, including C3 GN (MesGN)
B. Secondary GN was also classified into 9 groups:
1. SLE (Lupus nephritis),
2. Systemic vasculitis (with crescentic and segmental necrotising GN),
3. Other immune mediated (Goodpasture’s syndrome, cryoglobulinemic GN, scleroderma, Henoch-Schönlein)
4. Amyloidosis (AA, AL, Familial)
5. Monoclonal dysgammaglobulinemias (light-chain nephropathy, myeloma)
6. Diabetic nephropathy
7. Hereditary nephropathies (including Alport’s syndrome, Fabry’s )
8. HIV-associated nephropathy (HIVAN)
9. Others (including Fibrillary GN)
C. Tubulointerstitial disease (TID) including: Acute TID, Chronic TID, and Acute tubular necrosis (ATN).
D. Vascular nephropathies (VN) including thrombotic microangiopathies (TMA), Nephrosclerosis
E. Others (end-stage kidney, inadequate biopsy)

### 3.5. Ethical issues

The research followed the tenets of the Declaration of Helsinki. The research was approved by ethical committee of Lefkosa Burhan Nalbantoğlu State Hospital (State Hospital, Nicosia, North Cyprus).

### 3.6 Statistical analysis

Data were stored on a standard Excel database. The annual incidence was defined as the number of new cases per year related to the mean total population, expressed as per million population (pmp) per year.

## 4. Results

We reviewed 153 biopsies. The annual biopsy rate, averaged over the 10-year period (20016-2015), was 48 per million population (pmp) per year (see Table 2).

**Table 2:** Other Biopsy Series

**Country**			**Cyprus**		**Czech**	**Spain**	**Romania**	**Serbia**	**Italy**	**Italy**	**India**	**France**	**Finland**	**Australia**	**Australia**	**Denmark**	**USA**
**Citation**					[8]	[9]	[12]	[10]	[13]	[11]	[14]	[15]	[16]	[17]	[18]	[19]	[20]
Year					2004	2002	2013	2009	2007	2004	2011	2004	2008	2015	2001	1999	2006
Age group (y)			> 17		All	All	>18	>16	All	All	>10	20-59	All	All	All	All	All
Mean Age			45.7		x	x	41.9	39.1	42		32			48			
Biopsies pmp/yr			48		69.3	48	12.8	10.8	x		x	162	254	120	261		175
Primary GN		N	***%***	*pmp/yr*	*pmp/yr*	*pmp/yr*	*pmp/yr*	*pmp/yr*	*pmp/yr*	*pmp/yr*	*pmp/yr*	*pmp/yr*	*pmp/yr*	*pmp/yr*	*pmp/yr*	*pmp/yr*	*pmp/yr*
FSGS 1^o^		10	12	3.1	3.5	6.4	0.51	1.3					9 (inc MCD)	10.2	43	5.7	18
FSGS 2^o^		25	29	7.8	-	-						8 (inc Mes-GN)			-	
IgAN		20	24	6.3	11.2	7.9	0.63	0.85				25	50	14.1	86	-	21
MesGN		3	4	0.9	3.7	-		1.74					77	2.1		10.8	
MCD		4	5	1.3	4	4.8	0.17	0.54				8		2.9	11	7.3	2
MPGN		8	9	2.5	1.5	3.6	1.31	0.69				2		1.5	26	2.1	5
MN		15	18	4.7	3	6.2	0.74	1.3				6	14	6.5		4.8	10
**Sum**		85	100														
** **				26.6	32	28.9	3.79	6.93				53		46.2		39	
**Sum**	% of all Bx	85	56		60	55.8	56	64.2	71.6	70.7	69.1						
Secondary GN																	
Amyloid		3	7	0.9	1.4	3.3								1.9			
DN		7	17	2.2	1.5									8.4			
HSP		2	5	0.6	0.8												
RPGN		8	19	2.5	2.1	4.3								7.3	3.1		
SLE		22	52	6.9	3.2	5.6								6.9	3.5		7
Sum		42	100														
** **				12.8	13.8		2.36	2.68						20.5			
Sum	% of all Bx	42	27		25	20.4	35.1	25.1	18.4	23.8	18.2						
Tubulo-interstial nephropathy															
TID	% of all Bx	22	14		4.4		2.5	3	5.7	2.3	6.7		14.7				
				0.0			0.17	0.34									
Vascular nephropathy																	
VN	% of all Bx	1	1		3.4		2.5	4.2	4.3	3.2	3.2		1.8				
ESRF/ Others		3	2	0.9	4.6								17				
IgAN/Bx rate	%			14%	16%	16%	5%	8%				15%	20%	12%	33%		

Legend: Countries (refs 15-20) report glomerular disease only. FSGS, focal and segmental glomerulosclerosis; IgAN, IgA nephropathy; MesGN, mesangioproliferative GN; MCD, minimal change disease; MPGN, membranoproliferative (or mesangiocapillary) GN; MN, membranous nephropathy; DN, diabetic nephropathy; HSP, Henoch-Schönlein purpura; Vasculitis, crescentic GN associated with systemic vasculitis; SLE, lupus nephritis; TID, tubulointerstitial disease; VN, vascular nephropathy; ESRF, end-stage renal failure; N = number of patients; Pmp, per million population.

The mean age of the population studied was 45.7 years old (range 18-78). Eleven percent were aged 65 years or older. Overall the sex distribution was similar (male 51%), but when those with SLE were excluded, 60% were male. 12% of those biopsied had a known family history of renal disease.

The most common clinical indication for renal biopsy was AUA (44%), followed by NS (31%), acute nephritic syndrome (10%), chronic tubular kidney disease (6%), and AKI (7%) (Table 3).

**Table 3:** Renal diagnosis according to clinical phenotype

** Clinical phenotype and renal diagnosis **	** No.**	** % of phenotype **	** % of total **
Nephrotic syndrome			31%
MN	16	36
1o FSGS (primary FSGS)	10	23
DN	7	16
Amyloid	3	7
MCD	4	9
MPGN	4	9
SLE	4	9
	48	100
Nephritic syndrome			10%
HSP	2
Systemic vasculitis (ANCA+ve)	8
MPGN	1
SLE	4
	15	100
AUA			44%
FSGS 2^o^	25	36
SLE	14	20
IgA 100%	20	29
Mesangioproliferative GN	3	4
MCGN	2	3
TID	3	4
	67	100
Chronic tubular kidney disease			8%
TID	9
Vascular nephropathy (VN)	1
Other (ESRF)	3
	13
AKI			7%
ATN - TID	4
ATIN - TID	5
ATN plus DN	1
	10
	153		100%

Abbreviations: MN, membranous nephropathy; FSGS, focal and segmental glomerulosclerosis; DN, diabetic nephropathy; MCD, minimal change disease; MPGN, membranoproliferative (or mesangiocapillary) GN; SLE, lupus nephritis; HSP, Henoch- Schönlein purpura; RPGN, crescentic GN associated with systemic vasculitis; IgAN, IgA nephropathy; TID, tubulointerstitial disease; ATN, acute tubular necrosis; ATIN, acute interstitial nephritis; AKI, acute kidney injury; AUA, asymptomatic urinary abnormalities.

The most common histopathological category was primary GN (56%), followed by secondary GN (27%), and TID (14%) (Table 2).

The most common primary GN was 2^o^ FSGS (29%), followed by IgAN (24 %), membranous 18% and a further 11 patients with 1^o^ FSGS (12%). Thus all patients with FSGS represented 41% of primary GN (see Table 2).

Lupus nephritis (LN) accounted for 51% of all those with secondary GN, and next was diabetic nephropathy (DN) with 17% (see Table 2).

In all, there were 20 patients with diabetes, who were biopsied to see if they had an alternative diagnosis to DN. Eleven had the NS of whom seven had just DN, and four FSGS or minimal change. Six other diabetics presented with AUA of which we found secondary FSGS (5), IgA nephropathy (1). The final three had acute interstitial nephritis (2), and acute tubular necrosis plus DN (1). Thus only 8 of 20 (40%) had DN, and in the rest a non-diabetic pathology was the principal finding.

TID was diagnosed in 14% of all diagnosis (Table 1). Only one patient was classified as vascular nephropathy.

We also calculated the annual incidence of the different conditions (see Table 2). IgA nephropathy was 6.3 cases per million population per year (pmp/year). When expressed as a percentage of the annual biopsy rate, 14% of all biopsies showed IgA nephropathy.

## 5. Discussion

Most published renal biopsy series are difficult to compare as they lack standardisation of presentation.

### 5.1. Demography

Incidence of renal diagnosis should be expressed in terms of the population and the number of biopsies performed (16). Our annual biopsy rate was 48 pmp/year, which compares with a range of 11-260 pmp/year reported in other series (Table 2). The local biopsy incidence and, therefore, epidemiology of renal disease will depend to a large extent on the local biopsy criteria and enthusiasm. For example, whether or not those with microscopic haematuria, diabetes, tubular disease, ‘hypertensive nephropathy’ are biopsied. Countries such as Australia and France reporting high IgA incidence have high biopsy rates. If the incidence of IgA nephropathy is expressed as a percentage of the biopsy rate, we found overall a median of 16% of all biopsies, which was similar to our study with 13% of the biopsies showing IgAN (Table 2).

The mean age of our patients was 45.7 years but this is difficult to compare with others as some series include children and others have different age ranges (see Table 2).

### 5.2. Definitions: clinical renal syndromes

Before one can compare data, the terminology must be defined. When comparing series, the more specific the renal syndrome, the more useful the information obtained. For example, NS is unambiguous, if properly defined, whereas AUA and nephritic syndrome are always likely to overlap. In our series the nephritic group is probably under-represented and the AUA conversely increased, by a lack of clinical information to make always the distinction. To increase specificity, we suggest that in the future AUA could be subdivided into those with persistent microscopic haematuria (hematuric nephropathy) and those without.

We have used chronic tubular kidney disease without significant proteinuria to define a clinical group with a tubular phenotype.

### 5.3. Comparing data

When we compare data, we should first express the incident rate or prevalence in terms of the population under study as cases per million (10^6^) population per year (16). Our results are expressed in this manner in Table 2, and one can then directly compare the incidence of, say, IgA nephropathy with other series using similar methods.

A second way to compare data, is to use the clinical syndrome as the comparator or denominator and our data are shown in this way in Table 3. Using NS as an example, we can compare the frequency of primary and secondary forms of GN that cause NS. We found only three series that gave appropriate data to compare with ours. In Romania (12), Spain (9) and Serbia (10) primary GN causing NS was membranous (24%-39%), FSGS (13%-23%) and MPGN (14%-32%). Our results fall into these ranges.

Since NS is perhaps the one clinical syndrome that everyone would biopsy, we can use it as a comparator to see what percentage of the biopsies are performed for this reason. Not surprisingly this ranges from 54% of all biopsies in the series with low biopsy rates (10) to 10% in series performing over 100 pmp/year (16). In our series it was 31% of biopsies.

### 5.4. Histopathological diagnosis

Some histological diagnoses are so specific that the pathological term can be used as a clinical diagnosis, such as IgA nephropathy, minimal change, and membranous nephropathy. Whereas FSGS without NS, and mesangioproliferative GN (MesGN), have no meaning until the histological findings are matched with the clinical findings, immune staining, and EM findings and then specific diagnoses can emerge, such as Alport’s syndrome, C3 glomerulonephritis, thin basement membrane disease. A number of renal diagnosis can only be made with EM (such as thin basement membrane nephropathy).

In our analysis we separated those with histological diagnosis of FSGS into primary FSGS with NS, and secondary FSGS with variable proteinuria and normal serum albumin. This is important as the causes, treatment, natural history of the two groups are quite different.

### 5.5. Our series

In our own clinical practice, we commonly observe two renal syndromes, or clinical phenotypes, that are unusual compared with Western Europe. Firstly, there are patients with persistent but variable microscopic haematuria, variable renal insufficiency, often renal cysts, and late onset proteinuria with normal albumin/creatinine ratio until eGFR is <60 mL/min. There is commonly a family history in such patients suggesting autosomal dominant inheritance. Similar families, with *COL4A3/4* mutations, have been described in the Greek Cypriot population (3,21). The term hematuric nephropathy is often used to describe this group. In our population EM was rarely performed but in our series 23 patients with secondary FSGS and negative immune-staining, 61% of them had a haematuric nephropathy phenotype suggestive of a *COL4A*-like mutation. None had renal asymmetry to suggest renal scarring. So far in our population we have found two of the three *COL4A3/4* mutations originally reported in Greek Cypriots (3,22), plus one novel mutation. In addition, we have discovered a family with a mutation of *COL4A1* causing a non-syndromic haematuric nephropathy (6). Patients with *COL4A3/4* mutations are characterised by progressive FSGS, negative immune-staining and thin glomerular basement membrane (GBM) (21). We are investigating this population for further monogenic causes.

Secondly, we see many patients with a tubular phenotype who have little or no proteinuria (<1 g/d), variable chronic renal failure, sometimes renal cysts, but without hyperuricemia and gout. Families investigated with these tubular findings, including one of our own, have a linkage to the MCKD1 locus on chromosome 1q21 (4), and they have been subsequently identified as having a *MUC1* mutation (23). Vascular pathology consistent with long standing hypertension is often conspicuous in this condition even though hypertension is variable and easily controlled.

TID accounts for a median of 4% (1%-6.7%) in the series reported in Table 2. Our rate of 14% with an incidence of 6.6 pmp/year was higher. Acute injury was seen in 43% and 57% had chronic disease (Table 3), the latter possibly reflecting the commonness of patients with a clinical phenotype suggesting MCKD1.

## 6. Conclusions

For data to be compared between centres it must be expressed in terms of the population (incidence pmp/year) and the biopsy rate. In our population, secondary FSGS is common and currently many of this group are uncharacterised and will be caused by monogenic disease many of whom are expected to have mutations affecting the basement membrane proteins coded by *COL4A* genes.

## Limitations of the study

The main limitation of this study is the relatively small sample size. EM was available in only 18% of cases. Genetic investigation of those with secondary FSGS will be undertaken but results are not currently available.

## Acknowledgements

We thank Meral Yükseliş for assistance with data collection.

## Competing interests

The authors declare that they have no competing interests.

## Authors’ contribution

DDO was the renal specialist responsible for all clinical care of the patients whose biopsies are being reviewed. She was also responsible for the planning, preparation and writing of the manuscript. GHN was involved in the planning, preparation and writing of the manuscript.

## Funding/Support

This work was supported by funding from the European Renal Association and St Peter’s Trust.
